# Interferon–β Induces Hepatocyte Growth Factor in Monocytes of Multiple Sclerosis Patients

**DOI:** 10.1371/journal.pone.0049882

**Published:** 2012-11-14

**Authors:** Nicolas Molnarfi, Mahdia Benkhoucha, Kristbjörg Bjarnadóttir, Catherine Juillard, Patrice H. Lalive

**Affiliations:** 1 Unit of Neuroimmunology and Multiple Sclerosis, Division of Neurology, Department of Clinical Neurosciences, Faculty of Medicine, University Hospital of Geneva, Geneva, Switzerland; 2 Department of Pathology and Immunology, Faculty of Medicine, University of Geneva, Geneva, Switzerland; 3 Laboratory Medicine Service, Department of Genetics and Laboratory Medicine, Faculty of Medicine, University Hospital of Geneva, Geneva, Switzerland; Klinikum rechts der Isar der Technischen Universitaet Muenchen, Germany

## Abstract

Interferon-β is a first-line therapy used to prevent relapses in relapsing-remitting multiple sclerosis. The clinical benefit of interferon-β in relapsing-remitting multiple sclerosis is attributed to its immunomodulatory effects on inflammatory mediators and T cell reactivity. Here, we evaluated the production of hepatocyte growth factor, a neuroprotective and neuroinflammation-suppressive mediator, by peripheral blood mononuclear cells collected from interferon-β−treated relapsing-remitting multiple sclerosis patients, relapsing remitting multiple sclerosis patients not treated with interferon-β, and healthy volunteers. Using intracellular flow cytometry analysis, increased production of hepatocyte growth factor was observed in circulating CD14^+^ monocytes from patients undergoing long-term treatment with interferon-β versus untreated patients. Complementary *in vitro* studies confirmed that treatment with interferon-β induced rapid and transient transcription of the hepatocyte growth factor gene in CD14^+^ monocytes and that intracellular and secreted monocytic hepatocyte growth factor protein levels were markedly stimulated by interferon-β treatment. Additional exploration revealed that “pro-inflammatory” (CD14^+^CD16^+^) monocytes produced similar levels of hepatocyte growth factor in response to interferon-β as “classical” (CD14^+^CD16^−^) monocytes, and that CD14^+^ monocytes but not CD4^+^ T cells express the hepatocyte growth factor receptor c-Met. Our findings suggest that interferon-β may mediate some of its therapeutic effects in relapsing-remitting multiple sclerosis through the induction of hepatocyte growth factor by blood monocytes by coupling immune regulation and neuroprotection.

## Introduction

The recognition of multiple sclerosis (MS) as an inflammatory, demyelinating, and neurodegenerative disease of the CNS [1] emphasizes the necessity for therapeutic strategies to target inflammation and neurodegeneration simultaneously. [2] This can be accomplished with combination therapies or by applying molecules that are capable of targeting both pathogenic processes. In the search for single molecules combining these requirements, recent reports suggest that hepatocyte growth factor (HGF) is one such candidate [3], [4].

HGF has strong neuroprotective properties [3], [5] reported to enhance the survival and maturation of myelin producing oligodendrocytes. [6], [7] HGF also exerts anti-inflammatory effects through T cell bystander deviation and inhibition of antigen-presenting cell (APC) function. [8], [9] In animal models of demyelinating diseases, HGF was recently shown to confer protective immunoregulation, and to promote myelin repair in the absence of modulation of the immune system. [3], [4] Likewise, data from MS patient suggest that HGF may also potentially contribute to stimulation for remyelination [10].

Interferon–β (IFN–β) is a first-line treatment for relapsing-remitting (RR)MS [11], [12] shown to exert potent immunoregulatory effects on myeloid cells such as monocytes, [13] and likely to confer neuroprotection through secretion of neuroprotective factors, [14] including HGF by microglia. [6] These data suggest that, by combining immunomodulatory and neuroprotective effects, HGF may be a promising mediator of the clinical benefit of IFN–β treatment in RRMS.

Here we report that monocytes from RRMS patients exhibited a reduced ability to produce HGF when compared with healthy volunteers, and that monocytes from IFN–β–treated RRMS patients produced significantly higher levels of HGF. These findings provide new information regarding the mechanisms that mediate the therapeutic effects of IFN–β in RRMS.

## Materials and Methods

### Study Design

Twenty-seven RRMS patients fulfilling the 2005-revised McDonald Criteria [15] and 17 age– and sex-matched healthy individuals were recruited at the University Hospital of Geneva in accordance with institutional guidelines. All patients were classified as RRMS in remission for at least 3 months according to clinical history. Patients were required to be free of corticosteroid medication for at least 3 months before any blood sampling. All treated patients were receiving one single approved preparation of IFN–β–1a 44 µg 3 times/week subcutaneously (Rebif®, Merck Serono, Germany). Treated patients had to be on the same treatment regimen for at least 12 months. This study was performed using a single sample of venous blood. To minimize the possible acute effects of IFN–β, all samples were taken in the morning before the next scheduled injection, which was routinely self-administered in the evening. Characteristics of patients and healthy controls are described in Table 1. While pharmacokinetic information of IFN–β–1a on patients with RRMS has not been evaluated, the steady-state concentration of IFN–β–1b is usually between 40 and 80 IU/mL in patients receiving 8×10^6^ IU subcutaneously three times a week. [16], [17] Higher concentrations of IFN–β are known to be necessary to achieve effects on immune parameters *in vitro* [18].

**Table 1:** Clinical characteristics of healthy controls and MS patients.

Clinical Category	N	Age (years)mean; range	EDSSmean; range	Sex (F/M)
Healthy controls	17	37; 22–54	–	9/8
RRMS	15	38; 20–49	2; 1–4	10/5
RRMS-IFN-β	12	41; 20–60	1.7; 1–3.5	7/5

EDSS, Expanded Disability Status Score; RRMS, patients with relapsing-remitting multiple sclerosis; RRMS-IFN-β, RRMS patients treated with interferon- β1a.

### Standard Protocol Approvals, Registrations, and Patient Consents

This study received approval from the Geneva University Human Studies Committee Institutional Review Board for experiments using human subjects. The participants provided their written informed consent to participate in this study. The ethics committee approved this consent procedure.

### Cell Isolation and Culture Conditions

PBMCs were obtained by density gradient centrifugation of human peripheral blood over Ficoll–Paque (Pharmacia). Isolated PBMCs were resuspended in complete culture medium consisting of RPMI 1640 medium supplemented with 10% fetal bovine serum, 4 mM L–glutamine, 25 mM Hepes buffer, 50 U/ml penicillin, and 50 mg/ml streptomycin (all components were purchased from Life Technologies). For certain experiments, CD14^+^ monocytes and CD4^+^ T cells were further isolated using negative selection microbeads (EasySep). The purity of CD14^+^ and CD4^+^ T cells obtained by negative selection was routinely >90%, as determined by flow cytometry analyses, PBMCs, CD14^+^ monocytes or CD4^+^ T cells were cultured for the indicated time with or without IFN–β–1a alone.

### Flow Cytometry HGF

For analysis of HGF expression by monocytes from healthy controls and RRMS patients, 32 ml (4×8 ml) of venous blood was drawn into 3.2% buffered sodium citrate Vacutainer Tubes (BD) and processed within 2 h. Cells were stained with antibodies against CD14 and HGF or its IgG isotypic control, and analyzed on a FACSAria (BD Pharmingen) using CellQuest software (BD Pharmingen). For each sample, the geometric mean fluorescence intensity (Gmean) of specific HGF–positive cells was compared with that of isotype–matched control cells. The ratio was calculated as the Gmean for HGF–positive cells divided by the Gmean for isotype–matched control cells. To determine c–Met expression among different PBMC populations, cells were stained with antibodies against c–Met, CD4, CD8, CD14, CD16, CD19, CD56 and the respective isotype controls from the same manufacturer (BD Pharmingen).

### HGF Immunoassay

PBMCs and/or monocytes were activated for 24 h to 72 h with the indicated dose of IFN–β–1a. HGF production was measured in culture supernatants using ELISA (Quantikine, R&D). All experiments were performed with PBMCs or untouched monocytes obtained by negative magnetic–activated cell separation (MACS). Experiments are representative of PBMCs or monocytes isolated from at least three different blood donors. Alternatively, serum HGF level was determined from sodium citrate plasma (BD Vacutainer tubes, for 8 ml of blood). The results for ELISA assays are expressed as an average of triplicate wells±standard error of mean (S.E.M). SOFTmax ELISA plate reader and software (Molecular Devices Corporation) were used for data analysis.

### Quantitative Reverse Transcription Polymerase Chain Reaction

Monocytes were activated with IFN–β–1a (200,000 UI/ml) for the indicated times. Total RNA was isolated using the RNeasy micro Kit (Qiagen) and quantitative real–time duplex PCR analysis (ABI 7500; Applied Biosystems) was conducted after reverse transcription with the iScript cDNA Synthesis Kit (Bio–Rad). Primers and probes were obtained from Applied Biosystems. The levels of HGF mRNA expression were normalized with the expression of a simultaneously analyzed housekeeping gene (β–actin). All measurements were conducted in triplicate.

### Preparation of Cell Extracts

Monocytes were activated with 50,000 to 500,000 IU/ml of IFN–β–1a. At the indicated time, the activation was stopped and total cell lysates were prepared and subjected to Western blot analysis. Total protein content was determined using the Bio-Rad DC protein assay (Bio–Rad).

### Western Blot Analysis

Samples were loaded onto a 12% sodium dodecyl sulfate polyacrylamide gel (20 µg protein per lane). Proteins were transferred onto a nitrocellulose membrane (Bio-Rad). After blocking, the membrane was probed with a polyclonal anti–HGF antibody (1 µg/ml) (Santa Cruz) and further incubated with a secondary HRP–conjugated antibody (Amersham) in the blocking buffer. Immunoreactive HGF was detected using an ECL kit (Amersham). To re–probe the blot for the housekeeping control, the membrane was stripped and incubated with an anti–GAPDH antibody (Santa Cruz).

### Statistical Analysis

Mean and median fluorescence intensities were recorded for each subset of cells, as defined by regions in two–color scatter plots. The level of HGF expression was determined from frequency histograms, as the difference between the median channels of the specific antibody sample and the isotype control (specific median, sMed). Differences between medians of controls and patients were statistically analyzed by Mann-Whitney *U* test. Prism version 5.0c was used for all statistical procedures. Data are given as mean±SD and SEM.

## Results

### In Vitro Induction of HGF Release by Human PBMCs in Response to IFN-β

**Figure 1:**
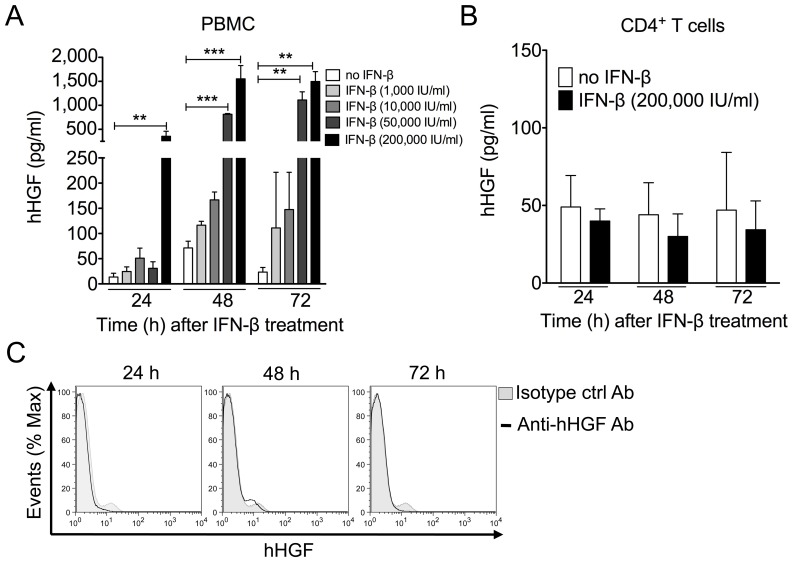
IFN–β induces HGF production by PBMCs. Human PBMCs or T cells were treated with IFN–β for the indicated time and dose. Culture supernatants were collected and HGF levels were evaluated using ELISA. (A) IFN–β increased release of HGF by human PBMCs in a time– and dose–dependent manner. (B) IFN–β did not stimulate HGF production by MACS–sorted CD4^+^ T cells. Data are expressed as means and standard deviations for triplicate wells of one representative experiment. **, *p*<0.01; ***, *p*<0.001, as determined by Student’s *t* test). (C) IFN–β did not induce cell–associated HGF levels by peripheral CD4^+^ T cells, as determined by flow cytometry. Cells were labeled with monoclonal anti–human HGF antibody or isotype control antibody and anti–human CD4 antibody. Representative histograms depict monoclonal anti–human HGF antibody (unfilled histogram) and isotype control antibody (filled histogram). Data are representative of three independent experiments.

We first evaluated the potential of PBMCs, particularly T cells, to secrete HGF in response to IFN–β. IFN–β induced a dose– and time–dependent increase in PBMC–secreted HGF, with the maximal effect obtained with 200,000 IU/ml of IFN–β at 72 h post–stimulation (Fig. 1A). In contrast, IFN–β did not induce release (Fig. 1B) or production (Fig. 1C) of HGF by CD4^+^ T cells, an important source of other neurotrophic factors (e.g., brain-derived neurotrophic factor, BDNF) in MS patients during immunomodulatory drug therapy.

### IFN-β Induces the Production and Release of Biologically Active HGF Protein by Monocytes

**Figure 2:**
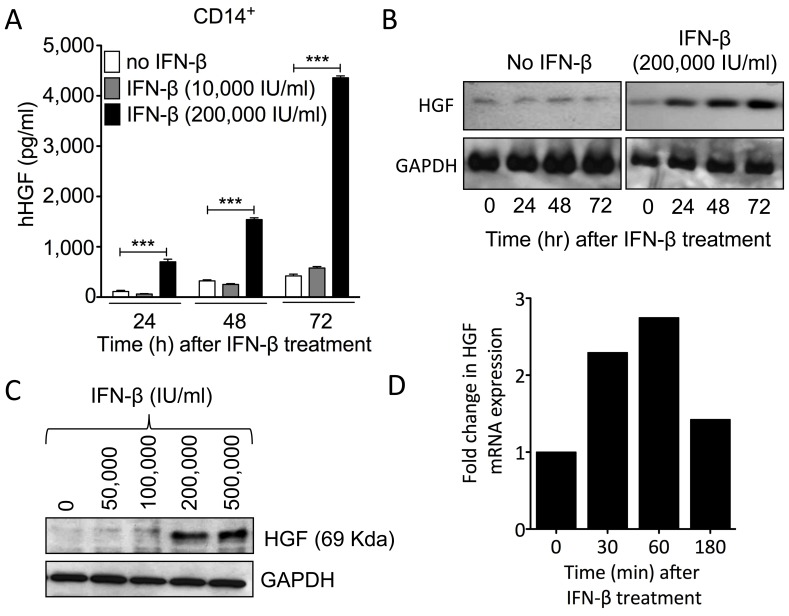
IFN–β stimulates *in vitro* HGF expression and the release of mature bioactive HGF by monocytes. (A) IFN–β increased HGF secretion by human MACS–separated monocytes from PBMCs in a dose-dependent manner, as determined by ELISA analysis. ***The mean value was significantly different from the control (medium alone) as determined by Student’s *t* test (*p*<0.0001). (B, C) HGF α–subunit (mature bioactive HGF protein) levels increased in monocytes in a time–dependent (B) and dose–dependent (C) manner in response to IFN–β treatment, as shown by Western Blot analysis. Cytosolic and plasma membrane proteins from MACS–separated monocytes were separated by SDS–PAGE and revealed using an anti–human HGF monoclonal antibody. The molecular mass of HGF is indicated. (D) IFN–β induces monocytic HGF gene expression, as determined by quantitative real–time PCR. Cytokine mRNA levels from MACS–separated monocytes were normalized with respect to the level of human β–actin. The results presented are representative of at least three different experiments.

We next investigated the role of IFN–β in HGF synthesis by monocytes, a primary cell target for immunomodulatory drugs in CNS autoimmunity. [19] Stimulation of monocytes with IFN–β resulted in a time–dependent release of HGF by the cells in the media, as determined by ELISA. A significant increase in HGF levels was observed as early as 24 h post-treatment, and was greatest at 72 h post–stimulation (Fig. 2A) (*p*<0.0001). Because this assay recognized both pro–HGF (inactive) and mature HGF, we next evaluated the ability of IFN-β to induce the production of biologically active (mature) HGF by monocytes. Western–blot analysis of the cell-associated fraction of IFN–β–treated monocytes demonstrated that HGF detected in monocyte supernatants was mainly in the cleaved mature form (presence of the 69–KDa α–chain) (Fig. 2B, C). Moreover, IFN–β induced HGF production in monocytes in a time– and dose–dependent manner (Fig. 2B, C). Using real–time PCR, we confirmed rapid gene expression of HGF by monocytes in response to IFN–β treatment, indicating that IFN–β induces HGF by activating its transcription (Fig. 2D).

### HGF Levels are Similar between CD14^+^ CD16^−^ and CD14^+^ CD16^+^ Monocytes

**Figure 3:**
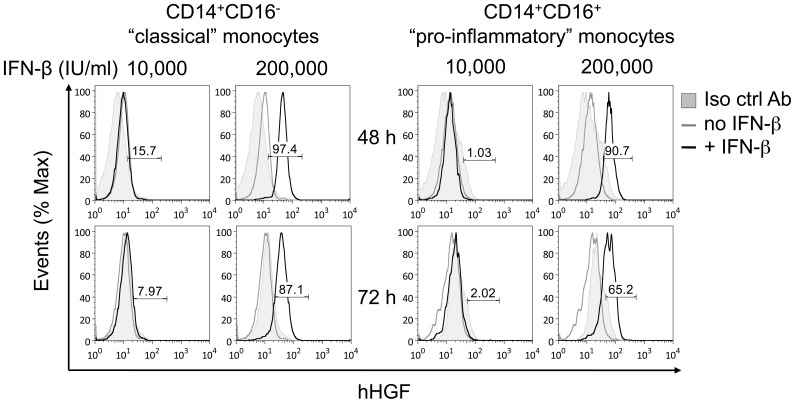
IFN–β increased cell–associated HGF in both human (A) CD14^+^CD16^−^ “classical” and (B) CD14^+^CD16^+^ “pro-inflammatory” monocytes in a time– and-dose–dependent manner. CD14^+^ monocytes were treated with IFN–β for the indicated time and dose, and cell–associated HGF was measured by flow cytometry. Cells were labeled with an antibody against anti–human HGF or isotype control antibody together with anti–CD14 and anti–CD16 antibodies. Histograms depict monoclonal anti–human HGF antibody (unfilled histograms) and isotype control antibody (filled histogram). Histograms depict representative data from two independent experiments. The values shown are the percentages of HGF positive cells as defined by fluorescence intensity greater than the control values of untreated cells.

With the use of anti–CD14 and anti–CD16 antibodies, human monocytes can be divided into two populations. Most cells are CD14 strongly positive (CD14^++^) and CD16 negative, representing what had previously been referred to as monocytes. Cells expressing CD14 at low levels together with the CD16 molecule (i.e., CD14^+^CD16^+^ monocytes) usually comprise 10% of all monocytes. With regard to their inflammatory phenotypes and functional properties in inflammation, CD14^+^CD16^+^ monocytes have been labeled proinflammatory. [20] Therefore, we compared HGF production by both monocyte subsets in response to IFN–β treatment by intracellular cytokine flow cytometry. IFN–β induced HGF expression in both monocyte subsets in a time– and dose–dependent manner with similar potency (Fig. 3A, B).

### HGF Receptor (c–Met) Is Expressed by CD14^+^ Monocytes and B Cells, but not T Cells

**Figure 4:**
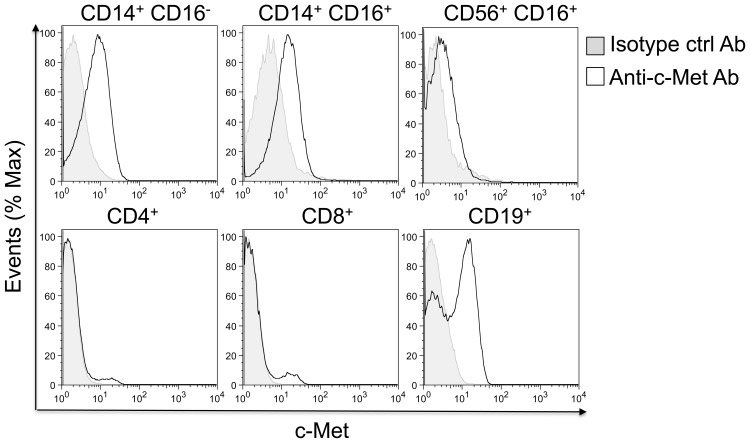
c–Met protein is expressed on CD14^+^ monocytes, CD19^+^ B lymphocytes, but not T lymphocytes. Surface expression of c–Met was evaluated on CD4, CD8, CD14, CD16, CD19, and CD56 PBMC subpopulations by six-color flow cytometry. Cells were labeled with monoclonal anti–human c–Met antibody or isotype control antibody and specific antibodies for CD4, CD8, CD14, CD16, CD19 and CD56. Histograms depict monoclonal anti-human c–Met antibody (unfilled histogram) and isotype control antibody (filled histogram).

For HGF production by monocytes to be of biological significance in immune reactions, the cells engaged in immune responses would need to express the HGF receptor. We used flow cytometry to test whether T and B lymphocytes expressed the high-affinity HGF receptor c-Met. c–Met is expressed by CD19^+^ B lymphocytes, but not CD4^+^ or CD8^+^ T cells, creating the potential for a functional interaction between monocytes and B lymphoid cells (Fig. 4). These studies confirm previous publications with other lymphoid tissues revealing strong c–Met expression by B cells, but not T cells. [21] Additional experiments revealed c–Met expression by both classical and pro–inflammatory CD14^+^ monocyte fractions of PBMCs. Finally, c–MET was expressed by CD56^+^ CD16^+^ natural killer cells (Fig. 4).

### Monocytes from IFN–β−Treated MS Patients Exhibit Increased HGF Production

**Figure 5:**
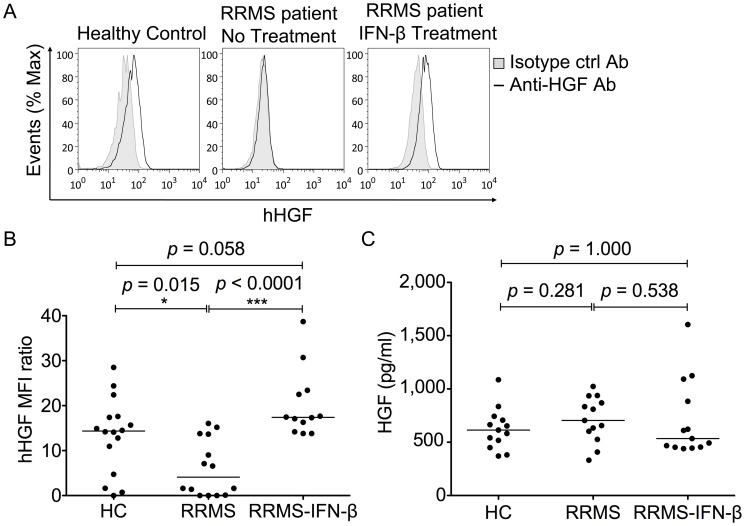
IFN–β–treatment increased levels of cell–associated HGF in monocytes from RRMS patients. (A) Flow cytometry for HGF was performed on CD14^+^ monocytes from the three groups (healthy control, untreated RRMS patients, and IFN–β−treated RRMS patients). Monocytes were stained for surface CD14 antigen and cell–associated HGF. Data show representative histogram overlays of isotype (filled histogram) and HGF–stained cells (unfilled histogram). (B) Cell-associated HGF levels in CD14^+^ cells were higher in healthy controls and IFN–β–treated RRMS patients. Surface expression was measured by flow cytometry and calculated as the mean corrected fluorescence index (MFI) ratio. Background HGF expression was assessed by measuring the fluorescence of cells incubated with a nonspecific isotype control antibody similarly labeled. The MFI for control anti–HGF antibody isotype staining was divided with the HGF MFI of monocytes. (C) Median serum HGF levels were similar in all three groups. ELISA for HGF was performed on sera from the three groups (healthy control, untreated RRMS patients, and IFN–β−treated RRMS patients). For both monocyte and serum HGF level analysis, each circle represents a single individual and the lines show the medians. Difference in median levels between groups was examined by Kruskal-Wallis test followed by Mann-Whitney *U* test due to a non-Gaussian distribution of values. *, *p*<0.05 and ***, *p*<0.001.

To study the effect of *in vivo* IFN–β therapy on monocytic HGF secretion in RRMS patients, we compared cell-associated HGF levels in CD14^+^ monocytes from RRMS patients treated with IFN-β to those of age– and sex–matched untreated patients (see Table 1). Monocytes from IFN–β−treated RRMS patients (n = 12) expressed significantly higher levels of HGF compared to those of untreated patients (n = 15) (Fig. 5A, B). We also evaluated the monocytic expression of cell-associated HGF in healthy controls, untreated RRMS patients, and RRMS patients treated with IFN–β (RRMS–IFN–β patients). Monocytes from healthy controls and RRMS–IFN–β patients exhibited significantly higher levels of HGF than monocytes from untreated RRMS patients (Fig. 5B). HGF was measured in the sera of IFN–β−treated and untreated patients, as well as healthy controls (Fig. 5C). Median serum HGF levels were not statistically different among the three groups (615 ng/ml (controls) vs. 705 ng/ml (RRMS) vs. 534 ng/ml (RRMS–IFN–β), *p* = *ns*).

## Discussion

In this study, we addressed the question of whether IFN–β treatment could induce the synthesis and secretion of HGF, a protein with immunoregulatory and neuroprotective properties, by PBMCs. To this purpose, HGF protein expression was analyzed in separate PBMC populations. We observed that IFN-β treatment induced strong HGF secretion by PBMCs, in particular by monocytes. Consistent with this observation, monocytes from untreated RRMS patients exhibit reduced HGF production compared with monocytes from IFN–β−treated RRMS patients. Therefore, *in vivo* IFN–β supplementation might prime monocytes to produce more HGF. HGF production may further restrain CNS autoimmune responses through autocrine/paracrine interactions with c–Met−expressing immune cells. Because HGF may be centrally involved in CNS autoimmunity, the induction of HGF production by IFN-β might contribute to the beneficial effects of IFN–β therapy in RRMS patients.

HGF is a pleiotropic cytokine that acts by binding to the HGF tyrosine kinase receptor, c-Met. HGF and c-Met are expressed within the immune system and their interactions have been shown to beneficially control immune cell functions in inflammatory animal models of disease in various organ systems, including the CNS. [3] HGF and c-Met are also expressed in brain-resident cells, including neurons, [22] mature oligodendrocytes, [7], [23] oligodendrocyte precursor cells (OPCs), [6], [23], [24] and microglia. [25] Consistent with its CNS localization, the functional coupling between HGF and c-Met was reported to enhance the survival of hippocampal neurons in primary culture and to induce neurite outgrowth during neuronal development *in vitro*. [26]–[29] HGF is as potent a survival factor for motor neurons as other survival factors, [5] such as BDNF and ciliary neurotrophic factor. [30], [31] HGF is also able to induce the proliferation and migration of OPCs, [6], [23], [24] as well as inhibition of the proapoptotic caspase-3 pathway in oligodendrocytes. [7] Therefore, it is suggested that HGF may be involved in the processes of neuroprotection, attenuation of oligodendrocyte degeneration, and/or remyelination, and that high HGF levels in cerebrospinal fluid (CSF) might be partially involved in the repair of white matter damage in demyelinating diseases, [32] and in particular, in the stimulation of remyelination in MS patients. [10] In support of this assumption, HGF, as a neurotrophic and neuroregenerative factor, has recently demonstrated therapeutic effects in mediating repair and functional recovery in animal models of MS, [4] spinal cord injury, [33] and amyotrophic lateral sclerosis [34].

IFN–β is a first-line therapy for RRMS. The efficacy of IFN–β in RRMS is likely to be related, at least in part, to its effects on the regulatory pathways that control T cell reactivity and the transfer of inflammatory cells across the blood brain barrier. [12] Aside from its well–known anti-inflammatory properties, neuroprotective mechanisms of action have been recently described for IFN–β. [35], [36] Studies have indicated that IFN–β induces the secretion of nerve growth factors by endothelial cells [37] and BDNF in PBMCs from RRMS patients. [14] In addition, we previously revealed that, similarly to transforming growth factor (TGF)–β1, a cytokine that favors the remission phase of RRMS, IFN–β led to HGF production by murine microglia. [6] Although the precise mechanism underlying the neuroprotective effect of IFN–β remains elusive, these studies suggest that the therapeutic potential of this drug is likely a result of its anti-inflammatory and neuroprotective properties. This paradigm was recently illustrated by studies from our laboratory and others demonstrating that HGF inhibits CNS autoimmunity by conferring dual protective immunoregulation and neuroprotection. [3], [4] As suggested by our current study, increasing HGF levels may thus be one avenue of combined neuroprotective and anti-inflammatory mechanisms leading to the clinical benefit of IFN–β in RRMS.

Circulating CD14^+^ monocytes can be divided into two functionally distinct subpopulations, among which the CD14^+^CD16^+^ subset is found to be augmented in various inflammatory diseases and has the potential to release high amounts of proinflammatory cytokines upon stimulation. In MS patients, the depletion of CD14^+^CD16^+^ monocytes by glucocorticoids (GCs) has been suggested to contribute to GC-mediated immunosuppression. [20] Here we report that both monocyte subpopulations expressed equal levels of HGF upon IFN–β treatment. In contrast with previous studies indicating an inability of CD14^+^CD16^+^ monocytes to produce anti–inflammatory or immunomodulatory cytokines, [20] the present data suggest this monocyte subpopulation is a potent source of HGF, a molecule that exerts strong anti-inflammatory and immunosuppressive effects in addition to its neuroprotective properties. As data indicate that the absolute and relative numbers of CD14^+^ monocytes, as well as the proportion of CD14^+^CD16^+^ cells among all monocytes, are unaffected by IFN–β therapy in RRMS patients, these findings further rule out the possibility that a specific subpopulation of monocytes, responsible for the global expression of HGF, is decreased or dysfunctional in untreated MS patients, but recovers after IFN–β treatment.

Mechanistically, these *in vitro* data demonstrate that IFN–β treatment directly elicits blood monocytes to release newly synthesized HGF. Although, and not necessarily contradictory, no differences in serum HGF were observed among IFN–β–treated patients, patients not treated with IFN–β, and healthy controls. One may therefore hypothesize that the absence of release of HGF in the serum may be associated with the capacity of blood monocytes from IFN–β–treated RRMS patients to release mature bioactive HGF directly in specific targets, such as patients’ damaged CNS. Data further indicate that the maturation of pro–HGF to biologically active HGF is a crucial limiting step in HGF signaling, and occurs through a tightly regulated proteolytic cleavage step. Pro–inflammatory conditions increase pro–HGF convertase activity in monocytes, and subsequently the release of mature HGF. Similar to HGF, data suggest that in IFN–β–treated MS patients, BDNF may be specifically delivered by PBMCs at the site of re–activation (i.e., within the CNS). [14] Finally, regarding data indicating that monocytes express the HGF receptor c–Met, [38] our results suggest that HGF induced by IFN–β–activated monocytes may amplify its effect via an autocrine loop. This possibility is particularly important given the capacity of monocytes to differentiate into myeloid macrophages and DCs in the peripheral system and microglia in the CNS, the three potent APC populations that are known to amplify immune responses into the CNS. In support of this assumption, HGF has been recently shown to limit CNS T cell responses by promoting tolerogenic DCs [3], [9].

In conclusion, we have provided evidence of a novel mechanism by which IFN–β controls human innate, and potentially, adaptive immunity by inducing HGF expression in monocytes. This mechanism is likely to limit inflammation and immunopathology during the course of the autoimmune response, and might contribute to the therapeutic effects of IFN–β in RRMS through dual but independent immunoregulatory and neuroprotective facilitation.
